# Intersubband absorption properties of high Al content Al_*x*_Ga_1−*x*_N/GaN multiple quantum wells grown with different interlayers by metal organic chemical vapor deposition

**DOI:** 10.1186/1556-276X-7-649

**Published:** 2012-11-26

**Authors:** He Hui Sun, Feng Yun Guo, Deng Yue Li, Lu Wang, Dong Bo Wang, Lian Cheng Zhao

**Affiliations:** 1 Department of Information Materials Science and Technology, Harbin Institute of Technology, Harbin, 150001, China; 2 Renewable Energy Laboratory, Institute of Physics, Chinese Academy of Sciences, P.O. Box 912, Beijing, 100190, China

**Keywords:** Quantum wells, Interface, Intersubband, TEM, PACS, 61.72.Lk, 61.05.cp, 68.37.-d, 61.72.uj

## Abstract

High Al content Al_*x*_Ga_1−*x*_N/GaN multiple quantum well (MQW) films with different interlayers were grown by metal organic chemical vapor deposition. These MQWs were designed to achieve intersubband (ISB) absorption in the mid-infrared spectral range. We have considered two growth conditions, with AlGaN interlayer and GaN/AlN superlattice (SL) interlayer, both deposited on GaN-on-sapphire templates. Atomic force microscopy images show a relatively rough surface with atomic-step terraces and surface depression, mainly dominated by dislocations. High-resolution X-ray diffraction and transmission electron microscopy analyses indicate that good crystalline quality of the AlGaN/GaN MQW layer could be achieved when the AlGaN interlayer is inserted. The ISB absorption with a peak at 3.7 μm was demonstrated in MQW films with AlGaN interlayer. However, we have not observed the infrared absorption in MQW films with GaN/AlN SL interlayer. It is believed that the high dislocation density and weaker polarization that resulted from the rough interface are determinant factors of vanished ISB absorption for MQW films with the GaN/AlN SL interlayer.

## Background

AlGaN/GaN multiple quantum well (MQW) structures grown on GaN/sapphire templates (GaN templates) have attracted much interest for intersubband (ISB) transition devices operating in the near-infrared and mid-infrared spectral ranges, such as photovoltaic and photoconductive GaN/AlN quantum well (QW) detectors [1] and electrooptical modulators [2], benefiting from the large conduction-band offset (1.75 eV) between GaN and AlN. In addition, material transparency in a wide spectral region (360 nm to 13μm for GaN) [3], large longitudinal-optical phonon energy (92 meV for GaN), and the rather heavy-electron effective mass (0.22 × *m*_0_ for GaN) also guarantee the realization of ISB transitions at room temperature. Short-wavelength ISB absorption has been reported by several groups in AlGaN/GaN MQW structures [4-7] and coupled QWs [8]; ISB absorption at telecommunication wavelengths has also been observed at room temperature in self-organized GaN/AlN quantum dots [9,10]. Until now, most of the progress was made by molecular beam epitaxy [11,12], which is best suited for growing sharp QW interfaces in nitride-based ISB devices because of its inherently low growth temperature and slow growth rate. By contrast, metal organic chemical vapor deposition (MOCVD) is the most economically feasible technique for the mass production of such ISB devices. Although many papers have been published on AlGaN/GaN MQW films grown by MOCVD, ISB devices employing the AlGaN/GaN MQW structure grown by MOCVD are few [13,14]. Therefore, further research and development by MOCVD are essential.

Obstacles still exist to hinder the development of AlGaN/GaN MQWs grown by MOCVD. There is a large lattice and thermal mismatch between the high Al content AlGaN/GaN MQW layer and the GaN templates. The biaxial tensile strain in the MQW layer accumulates in the process of the MQW layering, grows thicker, and finally leads to crack formation [15,16]. Moreover, the tensile strain will induce a large density of threading dislocations [17,18], a typical problem of III-nitride layers grown on foreign substrates (10^9^ to 10^10^ cm^−2^). Additionally, the Al(Ga)N/GaN interface is found to be unstable when it is pseudomorphically strained onto GaN. The alloyed interface is formatted under stress, especially growing at high growth temperatures by MOCVD [19,20]. This interface degradation in MQW structures affects the ISB absorptions. In order to overcome these problems, it is necessary to adopt some strain relaxation techniques for MQW structure growth.

In this paper, we inserted different interlayers in the MQW structure to solve these problems. High Al content Al_0.4_Ga_0.6_N/GaN MQW films were grown by MOCVD with different interlayers. The remainder of this paper is organized as follows. Initially, the detailed deposition process of the film growth is described, followed by the presentation of morphology, structures, and optical properties of MQW films. Finally, it provides the result that, with the insertion of different interlayers, MQW film strain relaxation differs. To get a good insight into the strain relaxation, we analyzed MQWs using different characterization techniques.

## Methods

All samples were grown on (0001) sapphire substrates by Thomas Swan MOCVD system (Thomas Swan & Co., Ltd., County Durham, UK). A 2-μm-thick GaN template was first grown with H_2_ atmosphere. Samples consisted of 20 periods of Al_0.4_Ga_0.6_N/GaN MQW structures and a 10-nm Al_0.4_Ga_0.6_N cap layer. The thicknesses of the Al_0.4_Ga_0.6_N barrier and the GaN well were 5 and 3 nm in MQW structures, respectively. Si doping in the GaN well layer was conducted with SiH_4_ at a doping level of 6 × 10^18^ cm^−3^. In order to relax the stress, two sets of interlayer structures were designed and prepared. A 20-nm Al_0.3_Ga_0.7_N interlayer was inserted between the AlGaN/GaN MQW layer and the GaN template in sample A, whereas four periods of GaN/AlN superlattice (SL) layers (AlN barriers, 3.6 nm; GaN wells, 1.4 nm) were employed for sample B as the interlayer. The interlayer inserted in samples A and B was coherent with the MQW layer in design. Growth parameters were the same for the AlGaN/GaN MQW layer and the cap layer in different samples.

High-resolution X-ray diffraction (HRXRD) measurements were performed with Bede D1 high-resolution X-ray diffractometer (Bede X-ray Metrology, Durham, England, UK). *ω*-2*θ* Scan of the (0002) X-ray reflection, *ω*-scan for the symmetric (0002), and skew symmetric (10–12) reflections of MQW films were utilized to characterize the crystalline quality of AlGaN/GaN MQW layers. Surface morphology was characterized by Pico Scan TM 2500 (PicoPlus, Tempe, AZ, USA) atomic force microscopy (AFM). Microstructures were studied using a JEOL 2010F high-resolution transmission electron microscope (HRTEM) (JEOL Ltd., Akishima, Tokyo, Japan) operated at 200 kV. All TEM samples were prepared following the standard procedure of grinding, dimpling, and ion milling until electron transparency is achieved. ISB absorption was investigated using a Bruker 120HR Fourier transform infrared spectroscope (BRUKER AXS GMBH, Karlsruhe, Germany) with a single transmission of p-polarized light incident at the Brewster's angle at room temperature.

## Results and discussion

**Figure 1:**
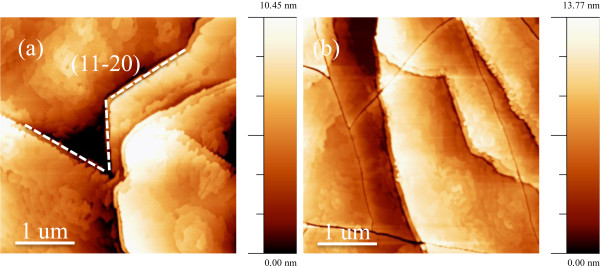
**AFM images of the samples with different interlayers.** (**a**) With AlGaN interlayer and (**b**) with GaN/AlN SL interlayer. The scanned area is 4 × 4 μm^2^.

Figure 1 shows the typical surface morphologies of AlGaN/GaN MQW films with different interlayers. A representative surface of sample A is shown in Figure 1a. It exhibited terraces separated by several bilayer high steps (1/2 c) that were obvious to resolve. In addition, undulations and deep pits, which appear to be arranged along lines, can be found throughout the surface. Regularly curved narrow trenches with preferred <10-10> crystallographic orientation were also observed throughout the surface. It is indicated that the surface was dominated by dislocation-mediated surface structures - spiral hillocks and surface depressions. Figure 1b shows the surface of sample B, in which terraces and undulations also exhibited on the surface, and moreover, micro-cracks were present on the surface. The value of root mean square roughness is shown in Table 1.

**Table 1:** **Surface roughness and FWHM of *ω*-scan for (0002) and (10–12) reflections of samples A and B**

**Sample**	**RMS surface**	**FWHM**			
		**(arc sec)**			
	**Roughness**	**MQW**		**GaN template**	
	**(nm)**	**<0002>**	**<10-12>**	**<0002>**	**<10-12>**
A	1.92	339	593	272.3	267
B	2.55	358	639	237	251

RMS, Root mean square.

**Figure 2:**
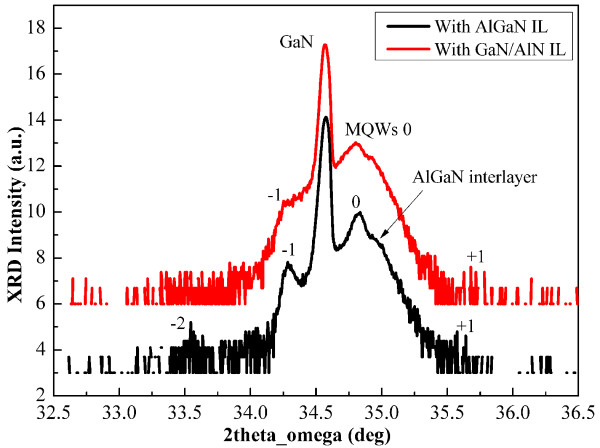
**(0002) *ω*-2*θ *scan of samples A and B.** IL stands for interlayer.

Figure 2 illustrates the (0002) *ω*-2*θ* scans of different samples. The clearly visible −2 to +1 order satellite peaks indicate a good periodical structure of MQW layers and sharp abruptness of AlGaN/GaN interfaces. For III-nitride films, the full width at half maximum (FWHM) of *ω*-scan for symmetric (0002) and asymmetric (10–12) reflections was usually adopted to access the threading dislocation density of MQW films and GaN template, respectively. The FWHM of *ω*-scan for GaN template and MQW films are shown in Table 1. Anomalously, the value of FWHM corresponding to MQW films with interlayers is larger than that of the GaN templates. When the interlayer was inserted, the FWHM increased, which seems to contradict the similar result of the traditional scheme [21,22]. Usually, the interlayer blocks the threading dislocation that originated from the GaN template, leading to the decrease of the FWHM of the subsequent film. Furthermore, it is found that the FWHM of the GaN template in sample B is lower than that in sample A; however, the FWHM of the GaN/AlGaN MQW layer in sample B is higher than that in sample A. The tendency is the same under different reflections. To explain the phenomenon, the FWHM broadening factors of *ω*-scan will be discussed in the following section.

FWHM of *ω*-scan will be broadened to the following, assuming Gaussian peak shapes [23]:

(1) $$\beta_{m}^{2}=\beta_{0}^{2}+\beta_{d}^{2}+\beta_{\alpha }^{2}+\beta_{\xi }^{2}+\beta_{L}^{2}+\beta_{r}^{2}\text{,}$$

where *β*_*m*_ is the measured FWHM, *β*_*0*_ is the intrinsic rocking curve width of the crystal, *β*_*d*_ is the instrumental broadening width, *β*_*α*_ is the broadening from lattice rotations at dislocations (tilt or twist), *β*_*ε*_ is the broadening due to lattice strain at dislocations (often called microstrain), *β*_*L*_ is the broadening due to limited correlation lengths, and *β*_*r*_ is the broadening due to wafer curvature. For *ω*-scan of symmetric (0002) and asymmetric (10–12) reflections, the FWHM broadening of zero-order satellite peaks mainly come from *β*_*α*_. When the threading dislocation density is close to 10^9^ cm^−2^, *β*_*L*_ cannot be neglected [23]. Furthermore, the interlayer in design is coherent with the MQW layer; the peak of interlayer and the MQW zero-order satellite peaks are overlapped in sample B, and the interlayer also contributes to the broadening of *ω*-scan. Therefore, the threading dislocation density of MQW films cannot be accessed directly by the FWHM of *ω*-scan.

**Figure 3:**
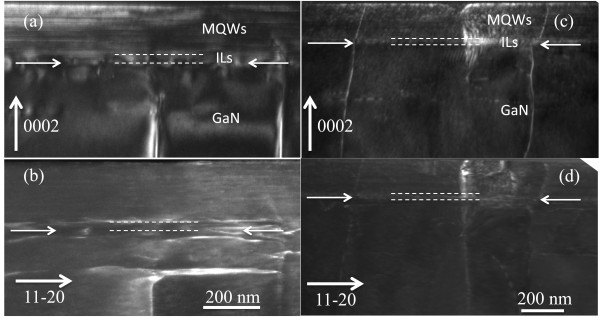
**Weak-beam dark-field images of samples A and B.** (**a, b**) The same region taken from sample A, using *g* vectors of (0002) and (11–20). (**c**, **d**) The corresponding images taken from sample B.

To access the dislocation density accurately, weak-beam dark-field TEM technique was taken. Images of samples are illustrated in Figure 3, with *g* = (0002) and *g* = (11–20) diffractions. Additional dislocations generated at the interface between the MQW layer and the GaN template in sample A (Figure 3a,b), except for threading dislocations that originated from the GaN template. Most of the dislocations are edge type, which is identified by the invisible criteria of *g* × *b* = 0. Threading dislocations, propagating from the interface between the AlGaN interlayer and the GaN template, were blocked by the AlGaN interlayer in sample A. Threading dislocations annihilated at the interface and were prevented from moving into the MQW layer. On the contrary, threading dislocations went into the MQW layer in sample B, which is shown in Figure 3c,d, without being blocked at the GaN/AlN superlattice layer interface. Threading dislocations just inclined and did not react with each other. The corresponding dislocation density for samples A and B are 6.4 × 10^8^ and 2.2 × 10^9^ cm^−2^, respectively. Without consideration of the influence of the GaN template's sample-to-sample variation, the difference of the threading dislocation density in the MQW layer is obvious.

**Figure 4:**
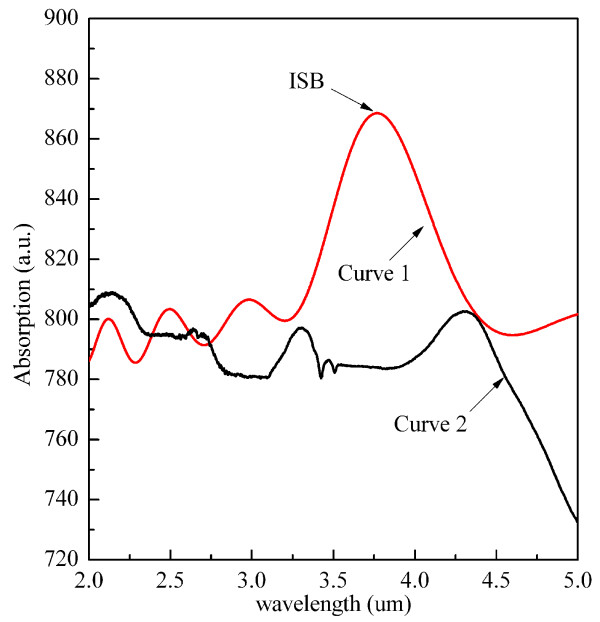
**The ISB absorption spectrum of samples A and B.** Curve 1 for sample A, curve 2 for sample B.

Figure 4 shows the representative ISB absorption spectrum for samples A and B. Curve 1 corresponds to sample A; the ISB absorption with a peak at 3.7 μm is obviously visible. Periodic interference peaks are also seen from curve 1, which resulted from the difference in refractive index between the epitaxial layer and sapphire substrate. However, no ISB absorption was observed in sample B (curve 2), except for periodic interference peaks.

In conclusion of Figure 3, the stress induced by the interlayer changes the propagation of dislocations. In sample A, the inserted AlGaN interlayer thickness exceeds the critical thickness of elastic relaxation. The AlGaN interlayer was plastic relaxed, as proven by the difference of peak locations in Figure 2. The tensile stress is strong enough to allow dislocations to meet, react, and annihilate. However, in sample B, the thickness of the GaN/AlN SL interlayer is the same as the AlGaN interlayer in sample A; the GaN/AlN SL interlayer is still under elastic relaxation, and the stress only makes dislocations inclined. Thus, dislocations incline and subsequently go into the MQW layer and finally make the dislocation density higher in sample B. Other stress relaxation paths are also present; cracks are shown in Figure 1b. Therefore, stress influences the propagation of dislocations as different interlayers were inserted. Details of dislocation behavior were discussed in another work [24]. From the results, it is manifested that the AlGaN interlayer effectively blocks the threading dislocation.

In sample B, threading dislocations entered the MQW layer, and no ISB absorption was presented in Figure 4. The absence of ISB absorption can be attributed to those reasons as shown below. First, the high density of threading edge dislocations exists in sample B. It was discovered that edge dislocations in III-nitride cause propagation loss for transverse-magnetic (TM) polarization of light in a waveguide [25]. Edge dislocations introduce acceptor centers along the dislocation lines, and electrons are captured by the acceptor centers [26]. Eventually, charges perpendicularly align and work like a wire-grid polarizer. This excess polarization-dependent loss is critical for ISB since ISB occurs only for TM polarization in a waveguide structure. Second, polarization charge in the GaN/AlGaN heterostructure will surely influence ISB intensity since ISB absorption strongly relies on the net carrier density in the QW. Compositionally graded AlGaN films grown on unintentionally doped GaN templates can achieve high electron-sheet concentrations which are results of polarization-induced doping [27-29]. According to the HRXRD analysis, order satellite peaks at −1 were present in sample B. No higher-order peaks manifested the rough interface in the MQW layer. As is well known, the electron density is crucial to the infrared absorption property of the ISB devices because electron–hole pairs cannot be generated only in the conductive band. The rough interface of the sample with SL interlayer surely decreased polarization-induced net electron density in the quantum well. Besides, interface instability pushes the first excited electron state close to the barrier conduction band edge. As a consequence, the e1 and e2 oscillator strength is reduced, and the ISB absorption becomes weak or even vanishes [30].

## Conclusions

In conclusion, high Al content AlGaN/GaN MQW films grown with different interlayers by metal organic chemical vapor deposition were investigated using AFM, HRXRD, and HRTEM. The MQW films present a relatively rough surface with atomic-step terraces and surface depression. Good crystalline quality of the AlGaN/GaN MQW layer could be obtained with the inserted AlGaN interlayer. The AlGaN interlayer under plastic stress effectively blocked the threading dislocation, but the GaN/AlN SL interlayer in the same thickness under elastic stress just inclined the threading dislocation. The ISB absorption with a peak at 3.7 μm in MQWs with the AlGaN interlayer, the high dislocation density, and the rough interface are the reasons of ISB absorption vanishing for MQW films with GaN/AlN SL interlayer.

## Abbreviations

AFM: Atomic force microscopy; AlN: Aluminum nitride; FWHM: Full width at half maximum; GaN: Gallium nitride; HRTEM: High-resolution transmission electron microscopy; HRXRD: High-resolution X-ray diffraction; ISB: Intersubband; MOCVD: Metal organic chemical vapor deposition; MQWs: Multiple quantum wells; SL: Superlattice; TM: Transverse-magnetic.

## Competing interests

The authors declare that they have no competing interests.

## Authors’ contributions

HHS carried out the experiments, measured the material, and drafted the manuscript. FYG and LCZ directed the experiments and the drafting of the paper. DYL and DBW participated in the growth of material process. WL was involved in revising the manuscript. All authors read and approved the final manuscript.
